# Agent tracking: a psycho-historical theory of the identification of living and social agents

**DOI:** 10.1007/s10539-014-9447-x

**Published:** 2014-04-29

**Authors:** Nicolas J. Bullot

**Affiliations:** ARC Centre of Excellence in Cognition and its Disorders, Department of Cognitive Science, Macquarie University, North Ryde, NSW 2109 Australia

**Keywords:** Agent, Animacy, Apparent agency, Misidentification error, Heuristics, Identification, Psycho-historical theory, Tracking, Mechanism

## Abstract

To explain agent-identification behaviours, universalist theories in the biological and cognitive sciences have posited mental mechanisms thought to be universal to all humans, such as agent detection and face recognition mechanisms. These universalist theories have paid little attention to how particular sociocultural or historical contexts interact with the psychobiological processes of agent-identification. In contrast to universalist theories, contextualist theories appeal to particular historical and sociocultural contexts for explaining agent-identification. Contextualist theories tend to adopt idiographic methods aimed at recording the heterogeneity of human behaviours across history, space, and cultures. Defenders of the universalist approach tend to criticise idiographic methods because such methods can lead to relativism or may lack generality. To overcome explanatory limitations of proposals that adopt either universalist or contextualist approaches in isolation, I propose a philosophical model that integrates contributions from both traditions: the psycho-historical theory of agent-identification. This theory investigates how the tracking processes that humans use for identifying agents interact with the unique socio-historical contexts that support agent-identification practices. In integrating hypotheses about the history of agents with psychological and epistemological principles regarding agent-identification, the theory can generate novel hypotheses regarding the distinction between recognition-based, heuristic-based, and explanation-based agent-identification.

In their search for generalisations and laws, *universalist* theories in the cognitive and brain sciences have posited mental mechanisms thought to be universal to all humans to explain agent-identification behaviours. Such ahistorical^1^ theories have paid little attention to how particular sociocultural and historical contexts interact with the mental processes involved in agent-identification. In contrast to universalist theories, *contextualist* theories appeal to particular historical and sociocultural contexts for explaining agent-identification. Contextualist theories tend to adopt methods aimed at documenting the heterogeneity of human behaviours across history, space, and cultures—an approach that is sometimes referred to as *idiographic*. To overcome explanatory limitations of proposals that adopt either universalist or contextualist approaches in isolation, I propose a philosophical model that integrates contributions from both traditions: the psycho-historical theory of agent-identification (“psycho-historical theory” henceforth). This theory investigates how the *tracking processes* that humans deploy for identifying agents interact with the unique *historical and sociocultural contexts* that support agent-identification practices. In integrating hypotheses about the history of agents with psychological and epistemological hypotheses about agent-identification, the theory can generate novel predictions regarding the distinction between recognition-based, heuristic-based, and explanation-based agent-identification.

## Universalist and contextualist theories of agent-identification behaviours

### The universalist approach

Let us first analyse a series of influential works that adopt the universalist approach. Fritz Heider’s research (Heider and Simmel 1944; Heider 1958) is an illustrative example of the universalist approach to agent-identification. Consider Heider and Simmel’s (1944) influential studies on the attribution of agency. The authors asked participants to watch a silent film showing the movements of two triangles and a circle that moved around and within a rectangular shape. In one of these experiments, the task given to the 34 participants was simply to “write down what happened in the picture” (1944: p. 245). Surprisingly, without any instructions to do so, all but one participant spontaneously described the three moving shapes as living agents or persons with mental states.

Heider and Simmel’s (1944) results support the hypothesis that humans have a propensity to interpret specific biomechanical patterns in terms of agency, even in cases where they do not believe that a real agent is present. Their studies played an important role in the development of Heider’s (1958) ahistorical *attribution theory* (see also Jones et al. 1971), which argues that the core concepts of common-sense psychology derive from a universal conceptual system for interpreting behaviour and attributing causal dispositions to agents and objects (1958: e.g., p. 14). Heider proposed that the experience of illusory agency elicited by Heider and Simmel’s inanimate stimuli was a consequence of the participants’ attribution of causal dispositions to persons understood as apparent causal origins (Heider 1944: pp. 359–362) and apparent causal units delineated by principles and “laws” of Gestalt psychology (1944: pp. 362–369; 1958: p. 22)—a form of *psychological constructionism*. Although Heider defends this strong form of constructionism, his model of common sense psychology implies that the attributive mechanisms that induce the illusory experience of agency are nonetheless routinely associated with the successful identification of *real* persons or living agents. However, surprisingly, Heider does not analyse the contexts in which humans can track real agents reliably. Nor does he discuss the problem of whether or not humans can track and identify agents reliably.

Ahistorical theories that combine universalist approach with constructionism as described in Heider’s attribution theory are pervasive in the psychological and brain sciences. Most prominently, the *modularist* and *mechanistic* approaches to agent-identification (e.g., Baron-Cohen 1995; Bruce and Young 1986), which posit that the architecture of the human mind is comprised of specialised mechanisms for recognising agents and attributing mental states, tend to combine universalist and constructionist assumptions. In these theories, the modules of agent-identification are commonly assumed to perform intuitive processes, which dual-process theories characterise as *Type 1* (Evans and Stanovich 2013). Type 1 processes are contrasted with *Type 2* processes, which are reflective processes typically dependent on higher order cognition and working memory.^2^ Several modularist theories interpret Heider and Simmel’s (1944) findings as evidence for “false-positive” detections carried out by an evolutionarily ancient, automatic, fast and parallel perceptual module that has evolved to use Type 1 processes to detect goal-seeking behaviours (see, e.g., Atran and Norenzayan 2004; Baron-Cohen 1995). These accounts conjecture that agent-identification modules are a feature of the universal architecture of the human mind, although they do not provide detailed biological and evolutionary evidence for explaining the module’s universality.

**Fig. 1:**
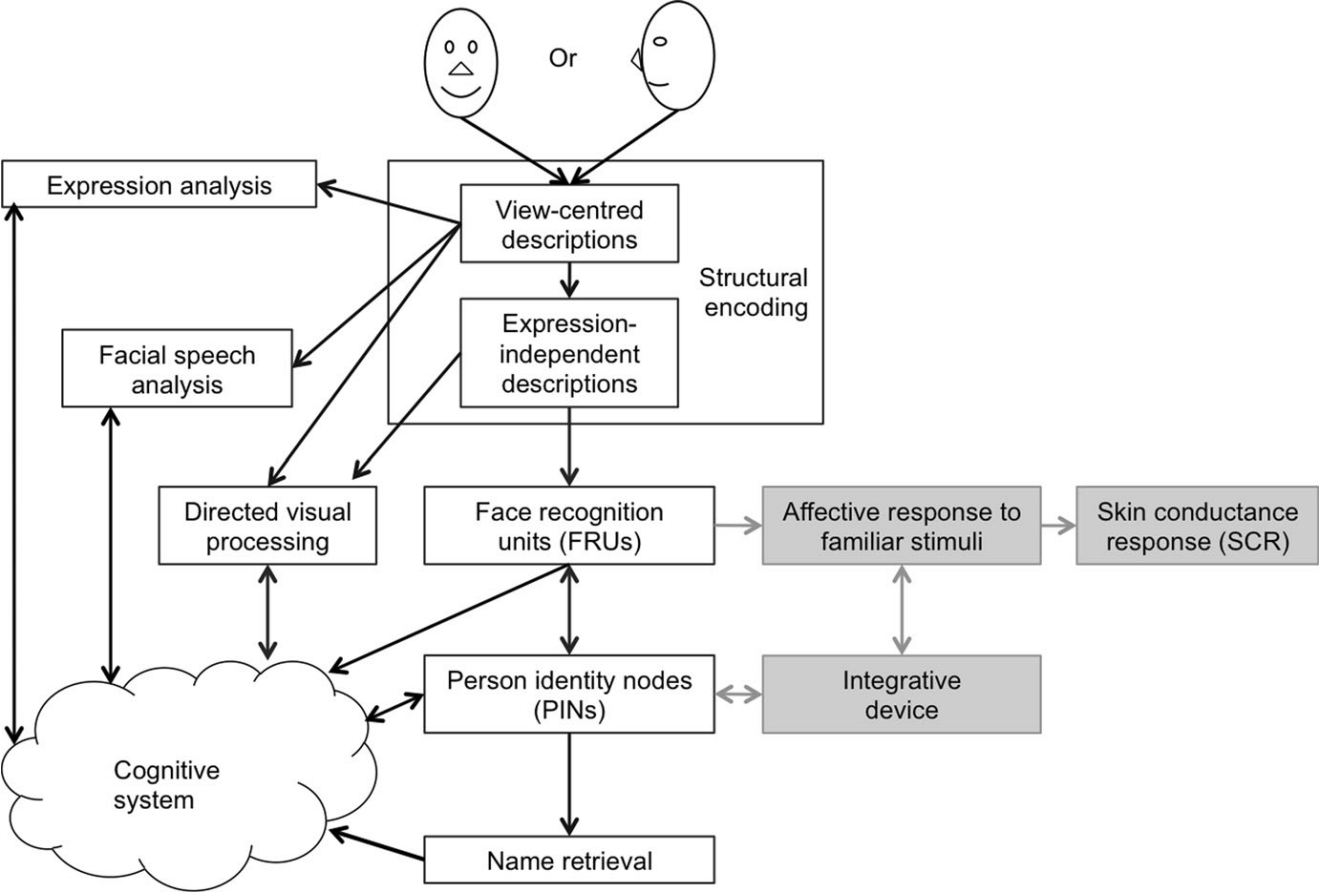
Bruce and Young’s (1986) model of person recognition and additional affective mechanisms proposed by Langdon (2011; see *boxes* and *arrows in grey shades*)

Bruce and Young’s (1986) model of face recognition (Fig. 1) is another example of an influential ahistorical theory that combines universalist and mechanistic hypotheses. This model seeks to explain *face*- and *person*-*identification* behaviours by decomposing the mechanism for face recognition into roughly four types of components and processes.

First, the recogniser’s exposure to diverse views of familiar faces triggers the structural encoding of invariant configurations of facial features. Second, face recognition units (FRUs) assess the familiarity of the input provided by structural encoding. Third, the detection of familiar faces by FRUs activates person identity nodes (PINs), which provide access to information about each person familiar to the perceiver. Fourth, the preceding processes enable retrieval of names. Other related models have highlighted the role of emotions in person-recognition—see boxes and arrows in grey shades in Fig. 1, which refer to mechanisms posited by Langdon (2011) and Gerrans (2012), among others.

The research programme developed by Bruce and Young (1986) and their followers examines neither the evolutionary history of person-identification mechanisms nor the interactions of such mechanisms with particular historical and sociocultural contexts. They assume that historical and sociocultural aspects of person-identification are factors that can be deemed negligible from standpoint of a purely psychological explanation.

The association between universalism and psychological constructionism is also found in other research programmes. For example, it is found in a number of prediction error theory of cognitive biases and perceptual illusions (see, e.g., Frith 2007: p. 140) and in recent clinical work, in which investigators have used the ability to experience illusory agents while perceiving Heider and Simmel’s stimuli as an indicator of normal social cognition (see, e.g., Horan et al. 2009).^3^

### The contextualist approach

In contrast to universalist theories that underappreciate the importance of context-specific processing, which are typically developed in cognitive psychology and neuroscience, *contextualist methods* for explaining agent-identification behaviours tend to dominate in the humanities and social sciences. These fields include the evolutionary anthropology of social learning (e.g., Richerson and Boyd 2005; Sterelny 2012), history (e.g., biographical history, see Davis 1983), theories of the techniques of person identification and tracking (e.g., Cole 2001; Nissenbaum 2010), and research on the identification artistic agency (e.g., Bullot and Reber 2013a).

Instead of considering contextual variability as a factor of negligible importance, contextualist theories take the variability of identification behaviours across historical and sociocultural contexts as a genuine *explanans*, a factor that should be cited to explain the *explanandum* phenomenon under consideration. For example, when discussing the social mechanisms involved in the forensic identification of recidivist criminals, a social scientist typically appeals to the sociocultural variability in methods for the forensic identification of persons in order to account for different patterns of identification behaviours. This is nicely illustrated by Cole’s (2001) detailed examination of the variability of performance in the identification methods (e.g., Bertillonage vs latent fingerprints analysis). Cole’s analysis is contextualist at least in the sense that it analyses a set of social mechanisms for person-identification that did not exist before a specific historical context: the context of British and French criminology during and after the nineteenth century. This type of explanation involves an *explanans* that accounts for its target *explanandum* by appealing to factors that are unique to a particular historical and sociocultural context.

### The puzzling antagonism between universalist and contextualist approaches

It is beyond the scope of this article to review the debates about the distinction between universalist and contextualist approaches, or nomothetic and idiographic methods. However, it is important to note that these debates have been controversial in several disciplines and often appear to be manifestations of the antagonism between scientific methods and scholarship in the humanities, the so-called “two cultures” debate (e.g., Bullot and Reber 2013b).

Defenders of the universalist approach have presented methodological objections to contextualist and idiographic approaches. They have argued that such methods lack generality and rigorousness, and can lead to relativism or scepticism about science—see, for example, Lamiell’s (1998) discussion of the psychological debates.

Advocates of contextualist and idiographic methods have argued that some universalist theories either fail to describe the variability of sociocultural phenomena (Ceci et al. 2010; Henrich et al. 2010) or tend to underappreciate the significance of intrinsically historical phenomena (for an early version of this argument, see Windelband 1894/1998).

Henrich et al. (2010) have challenged the methodology of the universalist approach and defended contextualism using cross-cultural research. Their challenge is based on the claim that universalist generalisations in the cognitive sciences are too often derived from narrow samples of human populations (Western, Educated, Industrialised, Rich, and Democratic—or “WEIRD”—populations) that, they argue, mask important patterns of variation. Because a number of universalist hypotheses appear false, they conclude that more empirical enquiries that take into account differences across cultural and historical contexts are needed to rigorously test the universalist hypotheses of ahistorical theories.

In regard to universalist theories that focus on agent-identification, those that rely on neuropsychological constructionism can inherit *epistemological* difficulties because they underestimate the importance of the distinction between representing fictive agents and identifying real agents in historical contexts. For example, Heider’s universalist theory of social attribution and the work of several of his followers raise an epistemological puzzle. Heider (1944) argued that processes generating the illusion of tracking agents from Heider and Simmel’s (1944) inanimate stimuli are involved in the tracking and identification of real agents. However, how could Heider’s constructivist hypotheses about the *same* mental mechanism explain *both* the *correct* identification of real agents and the *illusory* perception of non-existent agents (e.g., fictive persons imagined as a result of looking at Heider and Simmel’s stimuli)? To my knowledge, neither Heider’s theory nor subsequent studies in the psychological and brain sciences provide a framework for clarifying this fundamental epistemological puzzle.

## A psycho-historical theory of agent-identification

As an attempt to integrate explanatory elements from both universalist and contextualist theories, I propose a *psycho*-*historical theory of agent*-*identification* (hereafter “psycho-historical theory”) in the following sections. This theory expands the psycho-historical research programme proposed by Bullot and Reber (2013a, b) and Bullot (in press), which aims to describe relations of dependence between *context*-*specific* phenomena described by historical and social sciences and *context*-*sensitive* mental processes studied by the brain and behavioural sciences.

In contrast to the universalist models discussed above, the psycho-historical theory posits that psychological enquiry should be connected to a number of ontological and epistemological questions, which are considered negligible by universalist theories. For example, in respect to ontology, what is an agent or a person—that which is identified in agent-identification? In respect to epistemology, how do humans differentiate the reliable identification of an agent from erroneous and illusory cases of agent-identification? The psycho-historical framework provides a platform for integrating ontological and epistemological hypotheses that address these questions along with psychological questions about the mental mechanisms involved in agent-identification.

**Fig. 2:**
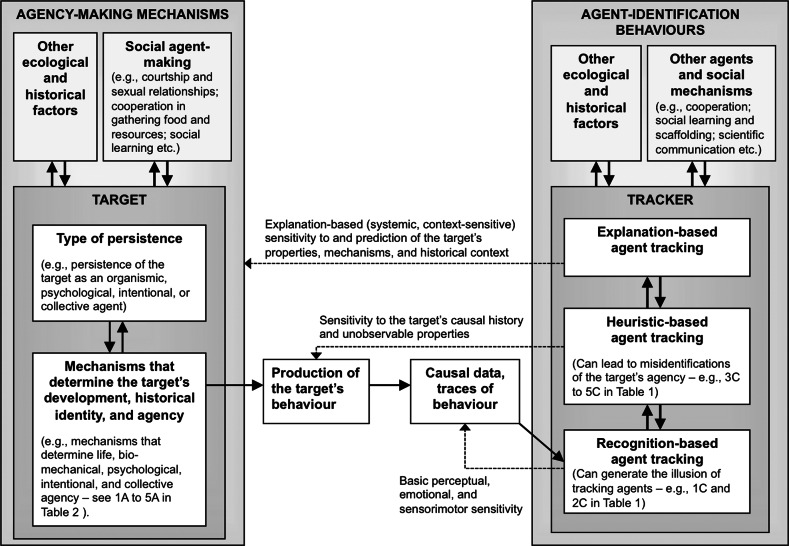
The psycho-historical theory of agent-identification: *solid arrows* refer to either causal-historical generation or feedback loops. *Dashed arrows* denote three types of tracking (sensitivity) derived from three types of identification processes

Figure 2 is a schematic of the main relations posited by the theory. The left-hand side represents an outline of the systems that determine the persistence, behaviours, and identities of agents. I term these systems *agency*-*making mechanisms*, and I discuss the different senses in which they belong to causal histories (“Ontological and historical components”).

The right-hand side represents the second component, introduced below: a *psychological* and *epistemological* model of the mechanisms involved in the tracking and identification of agents (*agent*-*identification mechanisms*). It aims to decompose *agent*-*identification behaviours* into three types of tracking abilities (i.e., recognition-based, heuristic-based, and explanation-based agent tracking). This component integrates evidence from psychological work on perceptual recognition, reasoning and heuristics, and episteomological work on folk and scientific explanation.

## Ontological and historical components

### The causal history of an agency-making mechanism

In contrast to universalist models of agent-identification in philosophy and psychology, the novelty of the psycho-historical framework is the inclusion of an ontological component that refers to the historical ontology of the target of agent-identification behaviours. This component is represented in the left-hand panel of Fig. 2. It illustrates the hypothesis that explanations of the persistence of biological and social agents need to refer to the causal histories of specific mechanisms, which are coined “agent-making mechanisms” in the theory. Although the majority of the examples I discuss pertain to the identification of humans, I assume that the theory can also provide working hypotheses to explain the identification behaviours that target non-human agents.

### Agents and the historicality of the tree of life

An important sense in which agents are historical entities comes from evolutionary biology. Evolutionary theories tell us that the ontology and lineages of living organisms can be productively described by the concept of the *tree of life*, “a network of ancestry and descent linking all organisms—all individuals as well as species—going back to a single root” (Godfrey-Smith 2009: p. 14). The tree of life hypothesis suggests that living individuals and species have core historical characteristics; for example, their existence is diachronic and each of them is the outcome of a *unique* historical network of ancestry and descent.

### Agents and their causal-historical interactions with environments

The historical nature of agents is also manifest from the fact that the development of a real living organism is the outcome of a *history of interactions* between the *internal parts of the developing agent* and physical, biological, or social *environments* (Oyama et al. 2001). For example, in addition to aspects of a physical environment necessary for life, the development of an organism is supported by a unique environmental niche (Laland et al. 2000) and, in social species, by a partly cooperative social environment (Sterelny 2012).

The two-way arrows at the left-hand side of Fig. 2 refer to the history of interactions between the individual agent and its environment. Analysing agent-environment interactions should not be restricted to the theories of morphological and psychological development. To explain complex cultural phenomena in agent tracking, such as the consequences of social sorting (Hacking 1986) and deception (see, below, the case of Arnaud du Tilh), it is also necessary to account for agent-environment interactions.

### The fundamental distinction between real (historical) agents and apparent agency

**Fig. 3:**
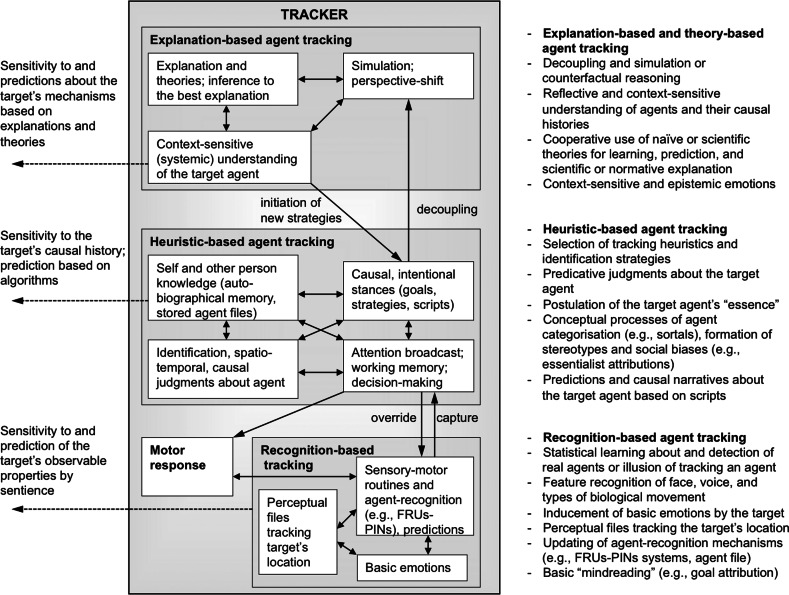
Decomposition of agent-identification behaviours into three types of tracking processes proposed by the psycho-historical theory. This figure expands the “tracker” component from the right-hand side of Fig. 2

In contrast to universalist theories of agent identification (see above), the historical ontology of the psycho-historical theory distinguishes the mechanisms that generate *real agents* (left-hand side of Fig. 2 and concepts 1A to 5A in Table 1) from the mechanisms that generate experience of and beliefs about *apparent agency* (right-hand side of Figs. 2, 3; and concepts 1C to 5C in Table 1). Table 1 identifies five possible concepts of real agency: the behaviours of living, biomechanical, psychological, intentional, and cooperative agency. According to the mechanistic approach of the theory, these different types of behaviour are likely to reflect the *causal histories* of different types of *agency*-*making mechanism*.

**Table 1:** Five kinds of real and apparent agency

	1. Living agency	2. Biomechanical agency	3. Psychological agency	4. Intentional agency	5. Cooperative (group) agency
A. Specification	1A. Property of mechanisms that are alive or are biological organisms (living agents)	2A. The property of having internal mechanisms that use the organism’s parts and sources of energy to control the organism’s locomotion and movements	3A. Property of mechanisms that have mental and psychological states such as desires, emotions, goals, intentions, perception or beliefs	4A. Property of mechanisms that cause intentional action, or have the disposition to do so	5A. Property of mechanisms and agents that cause collective coordination, cooperation, and collective action
B. Real, historical cases	1B. Living agents *qua* mechanisms involved in the development of living systems such as *genes*, *cells* and germ cells (Mayr 1982: Ch. 15), and *organisms* (e.g., Wilson and Barker 2007/2013)	2B. Non-human and human organisms, the movement of which can by described by *biomechanical models* (e.g., Alexander 2005)	3B. Non-human and human organisms that develop *mechanisms causing mental states/processes* such as emotions or proto-beliefs in primates (Sterelny 2003: Ch. 4)	4B. Non-human and human organisms that develop mechanisms causing or causation of motor acts by beliefs and desires (Davidson 1980/2001), or intentions	5B. Groups of agents that develop coordination mechanisms for *performing collective actions* (List and Pettit 2011)
C. Apparent, illusory cases	1C. Things that seem to be alive; stimuli that generate an ‘impression of life’ (Michotte 1946/1963) without being organismic; apparent living things, children’s animistic interpretations; attribution of life to certain artefacts (Guthrie 1993)	2C. Experience of animal locomotion in response to non-living artificial stimuli as in Michotte’s (1946/1963) caterpillar effect and other animistic interpretations	3C. Appearance of having psychological states or processes (Heider and Simmel 1944; Johnson et al. 2001)	4C. Illusory feeling of causing an intentional action. Mistaken attribution of individual agency, as in the attribution to Du Tilh of Guerre’s agency (“The difference between heuristic-based and explanation-based agent tracking”)	5C. “Free rider” agent (Sterelny 2012). Illusory appearance of group intentionality (Bloom and Veres 1999). Mistaken attributions of mental states and responsibility to aggregates and collectives (Heider 1958; Jones et al. 1971)

The universalist theories discussed above sometimes confound real and apparent agency. For example, what is loosely referred to as the perception of an “animate” in Heider and Simmel’s (1944) work and the subsequent literature may refer to either the perception of a real agent or the perception of an apparent agent. This ambiguous use, which can be prevented by adopting the distinctions presented in Table 1, might have contributed to the epistemological puzzle associated with constructionism in the psychological and brain sciences (see, above, “The puzzling antagonism between universalist and contextualist approaches”).

The types of *apparent* agency denote the experience of apparent agency that occurs in the absence of reliable tracking of real agents. For example, according to Guthrie’s (1993) theory of religion (see also the hypothesis of the hyperactive agent-detection devices: e.g., Barrett 2000), humans have a bias toward detecting human-like agency, which can elicit attribution of *apparent* agency to objects that do not have agency (i.e., “false positives”) in addition to successful detections (“hits”) in the detection of real agents.

This distinction between real and apparent agency might appear odd to readers who, like Heider and others, adopt the constructivist hypothesis that humans use the mechanisms that generate the illusions of apparent agency to track real agents. However, that worry can be dissipated by noting the importance of the distinction between tracking real versus apparent agents to explain errors in agent-identification (see, below, “Novelty of the psycho-historical theory” and “The difference between heuristic-based and explanation-based agent tracking”) and differentiate the agent-identification mechanisms, which is what I discuss next.

## Psychological and epistemological components

### Combining the mechanistic approach with contextualism

As a philosophical framework for explaining agent-identification behaviours, the psycho-historical theory combines a mechanistic approach to explanation with contextualist elements.

First, the theory adopt a *mechanistic approach* (e.g., Bechtel 2008; Craver 2007) suggesting a way to decompose a tracker’s agent-identification behaviour into specific *tracking mechanisms and processes*, such as those involved in recognising a target agent and reasoning about that target’s causal history. Although the theory does not fully specify the mechanisms it posits, it assumes that no principled reasons should prevent researchers from explaining agent-identification behaviours by decomposing them into tracking mechanisms.

Scholars have used concepts associated with the idea of *tracking a target across time* to describe various aspects of the identification of individuals and kinds.^4^ Here, I use the term “tracking” broadly to refer to mental and social processes that enable a subject, acting as a *tracker* (or *enquirer*), to *become sensitive* to causal processes that might have determined, currently determine, or might come to determine in the future the *persistence over time* and *behaviour* of an agent (the *target* 5 of the tracker’s act of identification).^6^ Examples are detailed below.

Second, the theory is *contextualist*; and it aims to complement universalist models of agent-identification with contextualist and epistemological hypotheses about agent-tracking. The theory is contextualist because it specifies the processes for tracking agents by reference to the tracker’s sensitivity to historical contexts and mechanisms. Moreover, it is also contextualist in that it posits that some agent-tracking processes can only occur in particular historical and sociocultural contexts, i.e., are *context*-*specific*. The bidirectional arrows between tracking types in Fig. 3 are aimed at suggesting that a number of tracking processes are “scaffolded” by collective and societal mechanisms (Bullot and Reber 2013b; Sterelny 2012; Sutton 2010), and that many tracking functions are performed by *groups* of human agents rather than individuals in isolation (see, below, “The difference between heuristic-based and explanation-based agent tracking”).

The psycho-historical theory posits that agent-identification behaviours comprise at least three types of tracking, based on recognition, heuristics, and inference to the best explanation. The corresponding tracking relations are represented by “sensitivity (dashed) arrows” in Figs. 2 and 3. Relying on an ontological model of the types of real and apparent agency (specified in Table 1 and Fig. 2), which delineates traceable and identifiable agents, the theory is an attempt to ascertain the limitations and epistemic power of each type of tracking.

### Perception-based and recognition-based tracking

Some processes enable a tracker to be sensitive to or predict *observable* properties of a target—including the tracker herself—without affording in-depth knowledge of that target’s unobservable causal properties and history. In the psycho-historical theory, these behaviours are classified as *perception*-*based* and *recognition*-*based* agent tracking (Figs. 2, 3). One of their plausible distinctive characteristics is that they can operate autonomously from higher order cognition, and therefore have the profile associated with Type 1 processing.

One of the most interesting Type 1 candidates is face recognition. Several sources of evidence indicate that the most basic forms of the *perceptual recognition of a face* and an agent can, as a Type 1 process, occur autonomously from the reflective reasoning about the causal history of the agent and possess a deep phylogenetic ancestry. First, psychological research conducted by Young et al. (1985) and Bruce and Young (1986), among others, provides evidence that a feeling of familiarity and identification are different processes. Such models distinguish the basic stages of face recognition that can trigger a *feeling of familiarity* (Bruce and Young 1986: p. 310)—or feeling of resemblance to a known person (Young et al. 1985: p. 517)—from the *decision*-*making process* supporting judgments about the identity of a person. This distinction corresponds to the distinction between recognition-based and heuristic-based agent tracking in the psycho-historical theory.

Second, independent empirical evidence suggests that recognition-based agent-identification can be controlled by phylogenetically ancient Type 1 processes. For example, ethological research has provided evidence suggesting that non-human species, especially social species, have capabilities for individual recognition (e.g., Cheney and Seyfarth 2007: Ch. 7; Tibbetts and Dale 2007). In ethology, *individual recognition* refers to a subset of recognition “that occurs when one organism identifies another according to its individually distinctive characteristics” (Tibbetts and Dale 2007: p. 529). Such agent-identification behaviours have been described in primates and other mammals like dolphins or koalas and several species of birds.

A great variety of perceptual and recognitional processes for agent-identification could fall into the category of Type 1 processes. For example, plausible Type 1 candidates include processes for the *implicit perceptual learning* of recurrent properties of an agent, perceptual recognition of the *kinematics* and *biomechanics* of an agent’s movements, recognition of the *face* and *sounds* signaling the presence of a particular agent, the *biasing of attention* towards agents and faces, and the triggering of *basic emotional responses* to an agent.

### Heuristic-based agent tracking

Although fundamental, recongition-based tracking probably cannot perform all agent-identification tasks reliably, most particularly those tasks in which the tracker has to predict and identify *unobservable* or *indiscernible* properties of the target.

An *argument from indiscernible persons and imposters* provide a rationale for thinking that agent-recognition cannot secure the *discriminative identification* of indiscernible agents (Bullot in press). Here, “discriminative identification” denotes the ability to discriminate a target agent from other persons that appear similar or indiscernible (e.g., Bullot and Rysiew 2007; Evans 1982).

The rationale of the argument is as follows. By most accounts, an important function of tracking mechanisms based on perceptual recognition is keeping track of a set of features that can serve to individualise a target agent. However, individual agents who come from a set of lookalikes, twins, and impersonators can present configurations of features that are practically indiscernible from one another. Therefore, one can predict that a basic human recognition-based tracking mechanism will not be reliable for tracking target agents from a set of indiscernible agents.

This point can be illustrated with Bruce and Young’s (1986) description of the mechanism integrating FRUs and PINs (Fig. 1). This mechanism encodes structural differences among faces taken as inputs to the mechanism for discriminating individual persons. Because the mechanism relies on visual appearances only, the mechanism alone does not have the discriminative power to differentiate perceptually indiscernible people who have different causal histories such monozygotic twins (e.g., Segal 1999/2000), accidentally similar persons (e.g., Will and William West^7^), and impersonators. Because its lacks contextual information about the distinct histories of indiscernible people, one can predict that the FRU-PIN mechanism will tend to generate “false positive” and “miss” errors when attempting to identify indiscernible people with distinct causal histories. This problem is likely to be shared by any recognition-based tracking mechanism that tracks targets over time merely on the basis of similarity and feelings of familiarity (Young et al. 1985).

Because recognition-based tracking alone is not sufficient to fulfil the task demands of agent-individualisation, the tracker may opt to engage in *explanatory stances* (i.e., strategies of enquiry; see Dennett 1987; Keil 2006) in order to better individualise the target by becoming sensitive to the target’s unobservable causal history and mechanisms (see Figs. 2, 3). In adopting such stances, the tracker may deploy strategies and heuristic principles aimed at inferring the target’s causal history and agency-making mechanisms from the target’s current behaviour or the traces left by the target’s earlier behaviour. In the *heuristic*-*based tracking* of an agent, searching and identifying an agent relies on judgments and strategies aimed at providing provisional causal narratives about the history, present, and future of the target. I use the term *heuristic*-*based* to refer to processes of identification that primarily operate by means of learned strategies and “shortcut” rules for facilitating judgment and decision-making (Gigerenzer and Todd 1999; Tversky and Kahneman 1974).

To illustrate the difference between basic recognition-based and heuristic-based tracking, consider the task of a tracker, Charles, who has undertaken to identify Clara and differentiate her from her *lookalike*, Lea, her *monozygotic twin*—so-called “identical” twin (Segal 1999/2000). To discriminate Clara from Lea, Charles needs to develop an ability to track pertinent aspects of Clara’s causal history and agency in order to discriminate her unique causal history from the causal histories of other similar individuals, especially Lea. In a number of circumstances, mere recognition-based tracking during exposure to Clara will not provide Charles with adequate sensitivity, because the similarity between the faces and voices of twins can induce considerable confusion (e.g., Sæther and Laeng 2008). To overcome this lack in sensitivity, Charles can resort to heuristics aimed at tracking subtle differences that monozygotic twin organisms accumulate, such as reversal effects (“mirror-imaging”) in their body structures (see Segal 1999/2000: pp. 22–25) and scars. Because these differences can be used for discrimination, a simple *twin*-*identification heuristic* consists in learning to recognise a feature that is distinctive of one of the lookalike-twins, searching for that unique feature, and ending the search when that feature has been recognised. Although I focused on distinguishing between identical twins, the “identification by unique-feature recognition” can be quickly deployed for individualising an agent in a variety of social contexts (if at least one its unique trait is known by the tracker). Furthermore, substituting a heuristic based on recognising a cluster of unique features can further improve this heuristic.

As illustrated by this example and Fig. 3, in *heuristic*-*based tracking*, the tracker’s adoption of a causal or an intentional stance specifies overarching goals and predictions, which in turn constrain specific search, attention-guidance, and recognition processes that subserve decision-making regarding agent-identification. Insofar as this heuristic-based tracking involves the endogenous guidance of attention broadcast, the use of working memory, and the updating of networks of semantic information and rules for agent identification (Renoult et al. 2012), heuristic-based tracking appears to have a Type 2 profile.

Multiple sources of evidence indicate that humans use heuristics for tracking agents in a variety of sociocultural and historical contexts; and that such heuristics are involved in both successful and erroneous cases of agent-identification. One source of evidence is that “fast and frugal” heuristics are quick and do not exceed the computational and memory requirements of the human mind (Gigerenzer and Todd 1999). Thus, as argued by Gigerenzer and colleagues, they can be helpful for the guidance of decision-making in contexts of time pressure, such as in legal and medical contexts. Identification heuristics are also likely to be of widespread use in circumstances when a tracker seeks to identify an agent who uses deception to evade identification, such as in legal reasoning (Hastie and Wittenbrink 2006), forensic enquiries, and the tracking of people on the Internet by means of data mining (Nissenbaum 2010).

Agent-identification heuristics may also provide the novice tracker with opportunities for “apprentice learning” in Sterelny’s (2012) sense: the cross-generational and cooperative transmission of environmental knowledge and social norms by expert agents to agents with less expertise. Identification heuristics could contribute to a tracker’s ability to learn skills and values shared within the community—such as learning the division of labour in social cooperation, the identification of trustworthy committed partners, the understanding of hierarchies of social control, and the ability to decipher the cultural identity of groups (2012: p. 49).

Finally, evidence for the use of heuristics also comes from research on biases induced by using heuristics. In the domain of agent-identification, several studies suggest that the tracking of persons’ identities can be biased by contextual information about the target person's social roles (Allen and Gabbert 2013), sometimes communicated by gossip (Anderson et al. 2011). Some of these biases might reflect a type of tracking that combines recognition-based tracking with heuristics.

In contrast to recognition-based tracking, heuristic-based tracking provide the tracker with an ability to simulate or infer *unobservable* *past and future states of the agent* from the agent’s behaviour and traces left by this behaviour (Figs. 2, 3). For example, if Charles knows causal facts about Clara’s history, Charles’ ability to differentiate Clara from her twin sister by means of the unique-feature heuristic will provide him with resources for simulating or inferring Clara’s past and future unobservable behaviour and the agency-making mechanisms that underlie her actions. Such an ability to become sensitive to unobservable facts will enable the tracker’s ability to *assemble narratives* about the history, unobserved present, and possible future of agents. Assembling and communicating narratives about the life of a person demands integration of the different tracking modes used to gather information about that person (Fivush et al. 2011). For example, Hastie and Wittenbrink (2006) report evidence indicating that jurors’ decision-making and identifications in court are driven by narrative-based accounts of the events under scrutiny.

### Explanation-based agent tracking

According to the psycho-historical theory, agent-identifications and predictions derived from either agent-recognition or heuristics can be outperformed by methods and techniques for tracking that benefit from scientific theories of the target’s agency-making mechanisms. This is because they equip the tracker with robust mechanistic models and predictions of the past and future behaviour of the target’s parts and agency-making mechanisms. Thus, simple heuristic-based agent tracking is, in principle, distinct from *explanation*-*based agent tracking* (see Fig. 2 and “The difference between heuristic-based and explanation-based agent tracking”). Explanation-based tracking corresponds to acts of pursuit and identification of an agent that derive from the tracker’s use of inferences to the best explanation (Lipton 1991/2004), which may include mechanistic explanations of the systems that cause the target’s persistence and agency.

The psycho-historical approach suggests that one should expect a close connection between tracking methods benefiting from explanations and explanation-generating theories that are the outcome of *context*-*specific* cooperative endeavours and techniques. For example, an unexpected outcome in a process of heuristic-based agent tracking may motivate a tracker to instigate novel explanatory strategies and produce inferences to the most likely explanation based on contrasting predictions made by different socially shared scientific theories. Such strategies would override recognition-based and heuristic-based tracking and take into account predictions derived from theoretical or scientific tools that are context-specific outcomes of social cooperation (Figs. 2, 3).

Theories can provide the tracker with means to better understand the relationships between (1) the alleged target’s historical agency and identity and (2) the underlying mechanisms that determine the target’s historical agency and identity. Such a mechanistic understanding provides the tracker with means to refine predictions about the target’s future behaviour or the target’s interactions with its environmental and social context, subsequently providing means to intervene in or control the target’s behaviour (Craver 2007).

## Novelty of the psycho-historical theory

In this section, I argue that the psycho-historical theory presents a number of novel hypotheses, arguments, and explanatory advantages when compared to either ahistorical universalist models or contextualist models that overlook biological and cognitive factors.

### Contrast with theories overlooking heuristic-based and explanation-based agent tracking

In contrast to the psycho-historical approach, universalist theories of agent-identification have not systematically investigated the *diversity* of tracking processes and social mechanisms involved in human agent-identification. Universalist theories have prioritised the study of recognition-based agent tracking over other types of tracking such as heuristic-based and explanation-based tracking. To the best of my knowledge, identifications of agents based on *context*-*specific heuristics and explanations* have not been studied systematically in the psychological and brain sciences. The psycho-historical theory provides a framework for expanding research programmes in these research fields.

### The social scaffolding of heuristic-based and explanation-based agent tracking

The psycho-historical theory proposes the novel contextualist hypothesis that *heuristic*-*based* and *explanation*-*based* tracking and identification are influenced by cultural and social *factors specific to the tracker’s historical and sociocultural context*. The theory suggests two predictions about heuristic-based tracking. First, socially scaffolded agent tracking is unlikely to be carried out solely by means of the Type 1 processing of recognition-based agent tracking. Second, because heuristics can induce biases and errors, the social scaffolding of agent tracking by socially shared heuristics can come at the cost of socially spread identification errors (see, below, the case of the misidentification of Martin Guerre) and social biases (e.g., social stereotyping).

By contrast, typical universalist models of agent-identification have not proposed this kind of hypothesis because they do not include a model of processing sensitive to historical and sociocultural contexts. For example, neither Heider’s attribution theory nor Bruce and Young’s (1986) model of face recognition encompass an *ontological* model of the target agent’s historical persistence and sociocultural context analogous to the left-hand part of Fig. 2.

### Historical feelings in agent-identification

Similarly to the psycho-historical theory of artefact appreciation (Bullot and Reber 2013a: p. 132, b: p. 169), the psycho-historical theory also suggests a hypothesis about historical feelings in agent-identification based on heuristics and explanations. I use *historical feelings* to refer to affective responses associated to the identification of an agent that are biased by the tracker’s knowledge about past historical and sociocultural contexts. For example, an experience of *nostalgia* triggered by the identification of a person who had been a close friend in the past would count as an historical feeling. Universalist theories have demonstrated that basic emotions such as fear (LeDoux 1996/1999) provide expressive signals and appraisals that can trigger or bias agent-identification (Gerrans 2012; Langdon 2011). However, because universalist theories of agent-identification have not systematically investigated the relations between historical feelings and agent-identification, the psycho-historical approach can be used to expand their research programmes.

### Epistemology of the tracking of an agent’s causal history

In contrast to universalist theories, the psycho-historical theory proposes novel *epistemological hypotheses* regarding the circumstances in which a human tracker can track herself and other agents reliably. A core hypothesis is that a process of tracking is reliable at identifying an agent if it can succeed in *re*-*tracing the causal history of the target agent* (either self or other). Reliability in agent-identification depends on the tracker’s ability to become sensitive to the causal mechanisms that determine the target’s persistence and causal history (agency-making mechanisms in Fig. 2). The psycho-historical theory also suggests that recognition-based tracking can be complemented with heuristic-based and explanation-based tracking to secure a tracker’s ability to reliably track or predict the unobservable past and future stages of an agent’s causal history.

The psycho-historical theory suggests a way to *classify misidentifications*. The mechanistic architecture of the theory implies that the propensity of each tracking mechanism to become sensitive to a target can be *impaired* by events such as damage to the mechanism (e.g., brain injury) or contextually inadequate use of the mechanism. This suggests that different types of agent-misidentification *errors* can be caused by dysfunctions of any of the three types of tracking posited by the theory.

## The difference between heuristic-based and explanation-based agent tracking

### Heuristics as a source of errors in agent-identification

Although identification errors can, in principle, be generated in any type of agent tracking, the psycho-historical theory posits that a crucial source of errors derives from heuristic-based tracking, which must therefore be distinguished from explanation-based tracking. The reader might be tempted to object that there is no principled distinction between heuristic-based and explanation-based agent tracking because both are likely triggered by explanatory stances, and run by simulation or reasoning. It is possible to rebut this objection by highlighting the epistemic limitations of tracking based on heuristics.

To further understand the specific *limitations of heuristic*-*based tracking* and justify its distinction from explanation-based tracking, it is useful to analyse, in some detail, a historical case of impersonation. Here I consider the remarkable case of Martin Guerre (Davis 1983; for a contemporary case, see Grann 2008).

Martin Guerre, a French peasant born around 1524 in Hendaye, left his wife, child, and village in 1548. In 1556, eight years after his disappearance, a man claiming to be Martin Guerre—call him “*New*-*Martin*”—arrived in the village. For about 3 years, New-Martin resided with Bertrande Guerre (Martin’s wife) and Martin’s son. After a complaint lodged by a relative, New-Martin was eventually suspected of impersonation and tried twice in court. During the final trial in which the genuine Martin Guerre made a surprising intrusion, Judge Jean de Coras (1561) and his confederates concluded that New-Martin was an imposter named Arnaud du Tilh (“*Arnaud*” henceforth). After this revelation Arnaud was sentenced to death for adultery and fraud and was executed on the 12th of September 1560.

What were the mechanisms that deceived the villagers? After an eight-year absence, the villagers’ ability to visually recognise Martin’s face might have become error-prone, which could have resulted in false-positives when they saw a similar face. On the other hand, it is also possible that the villagers may have disseminated mistaken identifications as a consequence of using error-prone heuristics. The analysis of historical sources (e.g., Davis 1983: p. 42, 79, 81, 84) suggests that the villagers—including Martin’s relatives—may have reasoned on the basis of heuristics such as this:

If a person who looks like Martin provides an accurate account of intimate autobiographical details of Martin Guerre’s past, then this person is [probably] Martin.

The villagers’ decision-making might have been influenced by socially “contagious” false beliefs (Lampinen et al. 2012; Zimbardo and Leippe 1991) and heuristics that take as a premise a judgement about a psychological trait deemed to be characteristic of Martin’s identity. Witnesses at the trials might have had recourse to a misleading inference conflating a mental *type* distinctive of Martin with the *particular causal history and mechanisms* that produced Martin’s agency (i.e., agency-making mechanisms in Fig. 2):

If that person who looks like Martin manifests attitudes such as beliefs, intentions, memories, values typical of Martin Guerre; then this person is [probably] Martin.

Although Martin’s wife should be an expert at identifying Martin, we can only propose conjectures regarding whether she was genuinely deceived by Arnaud or became Arnaud’s accomplice (Davis 1983). Other villagers might have relied on social learning strategies (Richerson and Boyd 2005), including *conformist inferences* about social relationships and the way people’s appearance changes. Such inferences provide simple heuristics and might reduce dissonance among potentially conflicting beliefs, as in:

If that person is recognised and accepted by Martin’s wife as her husband, then this person should be Martin in spite of conflicting accounts” (a *model*-*based bias* in the classification of Richerson and Boyd 2005: p. 69);

If most of our villagers identify that person as Martin, then this person should be Martin (a *frequency*-*based bias*).

The former heuristic-based inductive inferences are not necessarily truth-conducive. In the case of Arnaud’s impersonation of Martin, it is plausible that these heuristic-based inferences have been instrumental in producing the concatenated errors^8^ that made possible Arnaud’s creation of an *apparent Martin* for “free riding” Martin’s social network. Relying on the villagers’ use of heuristic-based tracking and acting in the manner of a “forger of agency”, Arnaud was successful at feigning important features of Martin’s agency such as Martin’s autobiographical memory, decision-making, and cooperative behaviour (concepts 3C–5C in Table 1: apparent psychological agency, apparent intentional agency, and apparent cooperative agency). Arnaud provided other agents with cues that were difficult to assess because they result from a Machiavellian dynamic, that is, he engineered a “*translucent* environment” (Sterelny 2003, 2012).

### Biases and errors generated by social heuristics

In line with research on biases induced by heuristics in statistical reasoning (Kahneman 2011; Tversky and Kahneman 1974) and social heuristics (Hastie and Wittenbrink 2006), the misleading heuristics in the identification of New-Martin illustrate how social heuristics can generate biases and errors in agent-identification. Relevant examples are not limited to social heuristics. For example, the “simple twin-identification heuristic” heuristic I discussed above (“Heuristic-based agent tracking”) will fail to provide a single tracker with the ability to individualise a target in a pair of twins or lookalikes if the criterion-feature is not uniquely inherent to the target (shared with another agent) or if it is concealed. Thus, although integrative research on agent-identification heuristics remains scarce (an important exception is Young et al. 1985), the frequency of agent-misidentification errors caused by heuristics is likely to be significant and have major societal implications—for example errors in distributions of social punishments or benefits as in the conviction of innocents in judicial trials (Lampinen et al. 2012).

The psycho-historical theory suggests a contextualist account of the detection and resolution of errors in heuristic-based agent tracking that requires the distinction between heuristic-based and explanation-based agent tracking. On this account, *errors* in heuristic-based agent-identification derive from the tracker’s *lack of sensitivity* to the history and agency-making mechanisms that cause the target’s persistence and behaviour (Fig. 2). In order to detect and overcome an identification error derived from the use of heuristics, the tracker needs *context*-*sensitive* methods that can outperform heuristics for *retracing the actual causal history* of the target and its agency-making mechanisms. Reference to these context-sensitive methods is missing in the ahistorical universalist models I have considered. The explanation-based tracking of an agent is context-sensitive in that sense because it can provide the tracker with models and theories of the target’s causal history, agency-making mechanisms, and historical context. It is only while using explanation-based tracking that the tracker opts to engage in strategies that aim to produce inferences to the best explanation, which draw inferences from the most likely or productive explanations of the target’s behaviour.

Explanation-based tracking has a Type 2 profile because it should demand that the tracker engages in the conscious reflection (i.e., metacognitive processes) about the outcome of other tracking mechanisms (Fig. 3). For example, the tracker may also benefit from becoming aware of the causal history of processes that led to the error. Moreover, following a number of accounts of inference to the best explanation, one can conjecture that inferences to the best explanation rely on an ability to consciously perform *contrastive reasoning* (Lipton 1991/2004). Contrastive reasoning is the ability to compare and adjudicate “facts and foils”, competing explanations from a pool of potential explanations, or competing hypotheses from a pool of empirical conjectures.

Heuristic-based identification is distinct from inferences to the best explanation because heuristics depend on the matching of a limited pool of criteria or rules rather than on insights provided by causal explanations or theories of the target’s intrinsic mechanisms. In contrast to explanation-based tracking, heuristic-based tracking has the profile of a “satisficing” method of decision-making, in which the decision-maker defines criteria for an aspiration level and ends the search for alternatives as soon as one that exceeds the aspiration level is encountered (Gigerenzer and Todd 1999: pp. 12–14; Simon 1990: pp. 9–10). By contrast to satisficing methods, inference to the best explanation is not tied to an aspiration level. Opting for explanation-based agent tracking provides the tracker with opportunities to use contrastive reasoning or simulations to compare and adjudicate competing explanations of the target’s identity and agency.

These considerations can be illustrated by the forensic discovery by the Judge Jean de Coras and his confederates that Arnaud du Tilh has impersonated Martin Guerre. The historical evidence available to us suggests that their final verdict (Arnaud’s conviction) resulted from explanation-based tracking. Specifically, there is evidence that the judges’ identification of Arnaud du Tilh as an impersonator should have resulted from contrastive reasoning aimed at providing the best explanation of the discrepant testimonies and narratives about Arnaud and Martin’s causal histories. Across the variety of contexts for tracking and reasoning based on contrasting different explanations, there is evidence that Coras used interventions and trickery to *contrastively* assess the epistemic value of the numerous testimonies (e.g., Davis 1983: pp. 77–78). For example, Coras manipulated the defendant’s and witnesses’ emotional responses to testimonies by means of confrontations of their conflicting accounts in the context of separate hearings (Davis 1983: p. 84), theatrical manipulations, and line-ups. The comprehensive explanation of Arnaud’s and Martin’s historical identities by all the parties was not the outcome of either tracking based on recognition alone or tracking based on heuristics alone. Rather, it was the outcome of a long and partially cooperative explanatory process aimed at retracing the causal histories of both Arnaud and Martin on the basis of a collective of trackers partially sensitive to the social mechanisms involved in the case and their own historical context.

At the beginning of the twenty-first century, contemporary trackers have access to a variety of scientific theories of agency-making mechanisms along with agent-identification techniques derived from such theories. A key oversight of universalist theories is that they do not account for the fact that the use of such resources by humans generates empirical evidence about biological, historical, and psychological persistence that were not available to Jean de Coras and his confederates. Had Coras’s enquiry benefited from relevant scientific explanations of the biological mechanisms that differentiate Arnaud and Martin’s organisms and link them to different causal genealogies, this forensic enquiry would have been greatly facilitated. For example, theories of inheritance mechanisms and DNA fingerprinting techniques (e.g., Pena and Chakraborty 1994) could have provided the forensic enquirers with means to assess the likelihood of the hypothesis that Arnaud du Tilh (Martin’s impersonator) is the father of Martin’s son. DNA fingerprinting would provide highly likely explanations that the impersonator is not the father’s of Martin’s son, and such biological assessment could have been combined with available sources of psychological and social evidence to undermine the impersonator’s claim, and precipitate the discovery of the imposture. This example illustrates that knowledge about identity-making and agency-making mechanisms can dramatically improve the reliability and predictive force of tracking and identification. It therefore demonstrates that actual processes of agent tracking are impacted by sociocultural factors and methods specific to particular historical contexts, a fact neglected by ahistorical universalist theories.

## Conclusion

Evidence gathered by universalist theories of agent-identification suggests that the basic recognition mechanisms for agent-identification are easily triggered by specific stimuli such as biomechanical patterns and face-like objects. However, this approach to agent-identification is far from providing a comprehensive explanatory framework for understanding the tracking of agents and persons. The more comprehensive account proposed by the psycho-historical theory is that, although agent tracking is typically triggered by mechanisms of perception, the most historically-sensitive and explanatory adequate modes of agent tracking should derive from integrating the outputs of several types of tracking mechanisms in heuristic-based and explanation-based agent tracking. By means of retrospective and predictive inferences, heuristic-based and explanation-based tracking use context-sensitive strategies that enable the tracker’s ability to identify unobservable facts and causal mechanisms that determine the persistence and agency of the tracked agent. As noted above, however, even processes of tracking by means of simple heuristics can lead a tracker to make systematic misidentification errors and confound an apparent agent with a real agent.

## Footnotes

1 I use the qualifier *ahistorical* to indicate that a theory does not include historical factors among its core explanatory constructs (see, e.g., Bullot and Reber 2013b). By *historical factors*, I mean variables and causal relations that are unique to, or distinctive to a particular era of evolutionary or human history.

2 Roughly, on Evans and Stanovich’s (2013) integrative account, the defining feature of *Type 1* (*intuitive*) processes are that they do not require working memory and are autonomous from higher order cognition. Typical but not necessary correlates of Type 1 processes include being fast, high capacity, parallel, nonconscious, automatic and associative. In contrast, *Type 2* (*reflective*) processes require working memory, cognitive decoupling and simulation.

3 Castelli et al. (2000) and Horan et al. (2009), among others, interpret the ability to perceive apparent animacy has an index of the normal functioning of theory of mind mechanism.

4 The terms *tracking* (e.g., Bullot and Rysiew 2007; Horowitz et al. 2007) and *tracing* (e.g., Rips et al. 2006) are often used to denote the ability to keep track of and identify a set of targets over a series of temporally distinct encounters.

5 Following Cummins (1996), I use *target* to refer to the entity that a tracking/identification mechanism has the function to track/identify.

6 Although I do not have space to discuss this point in detail, I assume that the ability to envision past and future episodes referred to as *mental time travel* (Suddendorf and Corballis 2007) is likely to play an important role in agent tracking.

7 The indiscernible appearances of Will and William West is allegedly what has caused the abandonment of the Bertillon system in favour of Galton’s fingerprinting system as a technique for forensic identification (see Cole 2001: pp. 140–142).

8 Concatenated errors are involved into major societal failure and disasters, see Reason (1990).

## References

[CR1] Alexander RM (2005). Mechanics of animal movement. Curr Biol.

[CR2] Allen R, Gabbert F (2013) Exogenous social identity cues differentially affect the dynamic tracking of individual target faces. J Exp Psychol Learn Mem Cogn. doi: 10.1037/a0033570 10.1037/a0033570 23815512

[CR3] Anderson E, Siegel EH, Bliss-Moreau E, Barrett LF (2011). The visual impact of gossip. Science.

[CR4] Atran S, Norenzayan A (2004). Religion’s evolutionary landscape: counter intuition, commitment, compassion, communion. Behav Brain Sci.

[CR5] Baron-Cohen S (1995). Mindblindness.

[CR6] Barrett JL (2000). Exploring the natural foundations of religion. Trends Cogn Sci.

[CR71] Bechtel W (2008) Mental mechanisms: philosophical perspectives on cognitive neuroscience. Routledge, New York

[CR7] Bloom P, Veres C (1999). The perceived intentionality of groups. Cognition.

[CR8] Bruce V, Young AW (1986). Understanding face recognition. Br J Psychol.

[CR72] Bullot NJ (in press) Explaining person identification: an inquiry into the tracking of human agents. Top Cogn Sci 10.1111/tops.12109 25124711

[CR9] Bullot NJ, Reber R (2013). The artful mind meets art history: toward a psycho-historical framework for the science of art appreciation. Behav Brain Sci.

[CR10] Bullot NJ, Reber R (2013). A psycho-historical research program for the integrative science of art [response to commentaries]. Behav Brain Sci.

[CR11] Bullot NJ, Rysiew P (2007). A study in the cognition of individuals’ identity: solving the problem of singular cognition in object and agent tracking. Conscious Cogn.

[CR12] Castelli F, Happé F, Frith U, Frith CD (2000). Movement and mind: a functional imaging study of perception and interpretation of complex intentional movement patterns. Neuroimage.

[CR13] Ceci SJ, Kahan DM, Braman D (2010). The WEIRD are even weirder than you think: diversifying contexts is as important as diversifying samples. Behav Brain Sci.

[CR14] Cheney DL, Seyfarth RM (2007). Baboon metaphysics: the evolution of a social mind.

[CR15] Cole SA (2001). Suspect identities: a history of fingerprinting and criminal identification.

[CR16] Coras JD (1561) Arrest memorable du parlement de Tolose, contenant vne hiftoire prodigieufe d’un supposé mari, aduenuë de noftre temps. Barthelemi Vincent, Lyon

[CR17] Craver CF (2007). Explaining the brain: mechanisms and the mosaic unity of neuroscience.

[CR18] Cummins R (1996). Representations, targets, and attitudes.

[CR19] Davidson D (1980/2001) Essays on actions and events, 2nd edn. Oxford University Press, Oxford

[CR20] Davis NZ (1983). The return of Martin Guerre.

[CR21] Dennett DC (1987). The intentional stance.

[CR22] Evans G (1982). The varieties of reference.

[CR23] Evans JSBT, Stanovich KE (2013). Dual-process theories of higher cognition: advancing the debate. Perspect Psychol Sci.

[CR24] Fivush R, Habermas T, Waters TEA, Zaman W (2011). The making of autobiographical memory: intersections of culture, narratives and identity. Int J Psychol.

[CR25] Frith CD (2007). Making up the mind: how the brain creates our mental world.

[CR26] Gerrans P (2012). Dream experience and a revisionist account of delusions of misidentification. Conscious Cogn.

[CR27] Gigerenzer G, Todd PM (1999). Simple heuristics that make us smart.

[CR28] Godfrey-Smith P (2009). Darwinian populations and natural selection.

[CR29] Grann D (2008) The chameleon: the many lives of Frédéric Bourdin. The New Yorker, 11 August http://www.newyorker.com/reporting/2008/2008/2011/080811fa_fact_grann

[CR30] Guthrie SE (1993). Faces in the clouds: a new theory of religion.

[CR31] Hacking I, Heller T, Sosna M, Wellbery D (1986). Making up people. Reconstructing individualism.

[CR32] Hastie R, Wittenbrink B, Gigerenzer G, Engel C (2006). Heuristics for applying laws to facts. Heuristics and the law.

[CR33] Heider F (1944). Social perception and phenomenal causality. Psychol Rev.

[CR34] Heider F (1958). The psychology of interpersonal relations.

[CR35] Heider F, Simmel M (1944). An experimental study of apparent behavior. Am J Psychol.

[CR36] Henrich J, Heine SJ, Norenzayan A (2010). The weirdest people in the world?. Behav Brain Sci.

[CR37] Horan WP, Nuechterlein KH, Wynn JK, Lee J, Castelli F, Green MF (2009). Disturbances in the spontaneous attribution of social meaning in schizophrenia. Psychol Med.

[CR38] Horowitz TS, Klieger S, Fencsik D, Yang K, Alvarez G, Wolfe J (2007). Tracking unique objects. Atten Percept Psychophys.

[CR39] Johnson SC, Booth A, O’Hearn K (2001). Inferring the goals of a nonhuman agent. Cogn Dev.

[CR40] Jones EE, Kanouse DE, Kelley HH, Nisbett RE, Valins S, Weiner B (1971). Attribution: perceiving the causes of behavior.

[CR73] Kahneman D (2011) Thinking, fast and slow. Farrar, Straus and Giroux, New York

[CR41] Keil FC (2006). Explanation and understanding. Annu Rev Psychol.

[CR42] Laland KN, Odling-Smee FJ, Feldman MW (2000). Niche construction, biological evolution, and cultural change. Behav Brain Sci.

[CR43] Lamiell JT (1998). ‘Nomothetic’ and ‘idiographic;: contrasting Windelband’s understanding with contemporary usage. Theory Psychol.

[CR44] Lampinen JM, Neuschatz JS, Cling AD (2012). The psychology of eyewitness identification.

[CR45] Langdon R, Calder AJ, Rhodes G, Johnson MH, Haxby JV (2011). Delusions and faces. Oxford handbook of face perception.

[CR46] LeDoux J (1996/1999) The emotional brain: the mysterious underpinnings of emotional life. Phoenix/Orion Books, London

[CR47] Lipton P (1991/2004) Inference to the best explanation. Routledge, London

[CR48] List C, Pettit P (2011). Group agency: the possibility, design, and status of corporate agents.

[CR49] Mayr E (1982). The growth of biological thought: diversity, evolution, and inheritance.

[CR50] Michotte A (1946/1963) The perception of causality. Methuen & Co., London

[CR51] Nissenbaum HF (2010). Privacy in context: technology, policy, and the integrity of social life.

[CR52] Oyama S, Griffiths PE, Gray RD (2001). Cycles of contingency: developmental systems and evolution.

[CR53] Pena SDJ, Chakraborty R (1994). Paternity testing in the DNA era. Trends Genet.

[CR54] Reason J (1990). Human error.

[CR55] Renoult L, Davidson PSR, Palombo DJ, Moscovitch M, Levine B (2012). Personal semantics: at the crossroads of semantic and episodic memory. Trends Cogn Sci.

[CR56] Richerson PJ, Boyd R (2005). Not by genes alone, how culture transformed human evolution.

[CR57] Rips LJ, Blok SV, Newman G (2006). Tracing the identity of objects. Psychol Rev.

[CR58] Sæther L, Laeng B (2008). On facial expertise: processing strategies of twins’ parents. Perception.

[CR59] Segal NL (1999/2000) Entwined lives: twins and what they tell us about human behavior. Plume, Penguin Group US, New York 10808244

[CR60] Simon HA (1990). Invariants of human behavior. Annu Rev Psychol.

[CR61] Sterelny K (2003). Thought in a hostile world: the evolution of human cognition.

[CR62] Sterelny K (2012). The evolved apprentice: how evolution made humans unique.

[CR63] Suddendorf T, Corballis MC (2007). The evolution of foresight: what is mental time travel, and is it unique to humans?. Behav Brain Sci.

[CR64] Sutton J, Menary R (2010). Exograms and interdisciplinarity: history, the extended mind, and the civilizing process. The extended mind.

[CR65] Tibbetts EA, Dale J (2007). Individual recognition: it is good to be different. Trends Ecol Evol.

[CR66] Tversky A, Kahneman D (1974). Judgment under uncertainty: heuristics and biaises. Science.

[CR67] Wilson RA, Barker MJ (2007/2013) The biological notion of individual. In: Zalta EN (ed) The Stanford encyclopedia of philosophy (Spring 2013 Edition) (pp. http://plato.stanford.edu/entries/biology-individual/). Metaphysics Research Lab, CSLI, Stanford University, Stanford, CA

[CR68] Windelband W (1894/1998) History and natural science. Hist Theory 8(1):5–22. doi:10.1177/0959354398081001

[CR69] Young AW, Hay DC, Ellis AW (1985). The faces that launched a thousand slips: everyday difficulties and errors in recognizing people. Br J Psychol.

[CR70] Zimbardo PG, Leippe MR (1991). The psychology of attitude change and social influence.

